# Superparamagnetic ZnFe_2_O_4_ Nanoparticles-Reduced Graphene Oxide-Polyurethane Resin Based Nanocomposites for Electromagnetic Interference Shielding Application

**DOI:** 10.3390/nano11051112

**Published:** 2021-04-25

**Authors:** Raghvendra Singh Yadav, Anju, Thaiskang Jamatia, Ivo Kuřitka, Jarmila Vilčáková, David Škoda, Pavel Urbánek, Michal Machovský, Milan Masař, Michal Urbánek, Lukas Kalina, Jaromir Havlica

**Affiliations:** 1 Centre of Polymer Systems, University Institute, Tomas Bata University in Zlín, Trida Tomase Bati 5678, 760 01 Zlín, Czech Republic; deswal@utb.cz (A.); jamatia@utb.cz (T.J.); kuritka@utb.cz (I.K.); vilcakova@utb.cz (J.V.); dskoda@utb.cz (D.Š.); urbanek@utb.cz (P.U.); machovsky@utb.cz (M.M.); masar@utb.cz (M.M.); murbanek@utb.cz (M.U.); 2 Materials Research Centre, Brno University of Technology, Purkyňova 464/118, 61200 Brno, Czech Republic; kalina@fch.vut.cz (L.K.); havlica@fch.vut.cz (J.H.)

**Keywords:** sonochemical synthesis, spinel ferrite, nanoparticles, nanocomposites, electromagnetic interference shielding

## Abstract

Superparamagnetic ZnFe_2_O_4_ spinel ferrite nanoparticles were prepared by the sonochemical synthesis method at different ultra-sonication times of 25 min (ZS25), 50 min (ZS50), and 100 min (ZS100). The structural properties of ZnFe_2_O_4_ spinel ferrite nanoparticles were controlled via sonochemical synthesis time. The average crystallite size increases from 3.0 nm to 4.0 nm with a rise of sonication time from 25 min to 100 min. The change of physical properties of ZnFe_2_O_4_ nanoparticles with the increase of sonication time was observed. The prepared ZnFe_2_O_4_ nanoparticles show superparamagnetic behavior. The prepared ZnFe_2_O_4_ nanoparticles (ZS25, ZS50, and ZS100) and reduced graphene oxide (RGO) were embedded in a polyurethane resin (PUR) matrix as a shield against electromagnetic pollution. The ultra-sonication method has been used for the preparation of nanocomposites. The total shielding effectiveness (SE_T_) value for the prepared nanocomposites was studied at a thickness of 1 mm in the range of 8.2–12.4 GHz. The high attenuation constant (α) value of the prepared ZS100-RGO-PUR nanocomposite as compared with other samples recommended high absorption of electromagnetic waves. The existence of electric-magnetic nanofillers in the resin matrix delivered the inclusive acts of magnetic loss, dielectric loss, appropriate attenuation constant, and effective impedance matching. The synergistic effect of ZnFe_2_O_4_ and RGO in the PUR matrix led to high interfacial polarization and, consequently, significant absorption of the electromagnetic waves. The outcomes and methods also assure an inventive and competent approach to develop lightweight and flexible polyurethane resin matrix-based nanocomposites, consisting of superparamagnetic zinc ferrite nanoparticles and reduced graphene oxide as a shield against electromagnetic pollution.

## 1. Introduction

Recently, the rapid progress in electronic devices and information technology has endorsed the widespread utilization of high-power electromagnetic waves in scientific, commercial, civil, and military applications [1,2]. The abundant electromagnetic interference (EMI) in the environment has influenced the working of electronic devices [3]. Exposure to electromagnetic radiation has also influenced human health [4]. Electromagnetic shielding or absorption has been demonstrated to be one of the operative approaches to address electromagnetic pollution [5]. Therefore, electromagnetic shielding or absorption material is one of the best procedures of environment and health defense [6]. A lightweight, flexible, and cost-efficient advanced nanocomposite shielding material is required to attenuate the detrimental electromagnetic wave interference [7].

The EMI-shielding mechanism consists of mainly the two-loss factors: (i) reflection loss and (ii) absorption loss. A single component, i.e., an only electric conductive material or magnetic material, has less competence to pay both the mechanism of reflection and absorption. The absorption loss-dominant shielding material is more desirable because it can prevent the second reflection pollution in comparison with the reflection-dominant material. In recent years, researchers and academicians have comprehended that the intention of nanocomposites consisting of both electric and magnetic constituents is a prudent choice to cultivate proficient EMI-shielding material [8]. Such deliberate shielding material is anticipated to close the gap between permittivity and permeability. In recent times, carbon-based materials, including carbon nanotubes, nanofibers, and reduced graphene oxide, have established significant consideration for efficient electromagnetic interference shielding material [9]. Reduced graphene oxide parades a high specific surface area with plenteous functional groups and structural defects on its surface. Therefore, it can deliver copious interfacial polarization and defect dipole polarization [10]. Reduced graphene oxide also exhibits outstanding dielectric characteristics and higher electrical conductivity [11]. In recent years, it is also perceived that the introduction of magnetic nanoparticles, including spinel ferrite in the presence of reduced graphene oxide, can moderate its impedance matching condition and robust attenuation capability of the shielding nanocomposite [12]. The microwave absorption characteristics also depend on the fraction of nanoparticles and reduced graphene oxide [13]. Further, superparamagnetic spinel ferrite nanoparticles have established a broad application due to their competence to hastily respond to an applied magnetic field, which is concomitant with negligible remanence and coercivity [14,15]. For that reason, there is an intensive research subject to develop superparamagnetic spinel ferrite nanoparticles and further their application as electromagnetic interference shielding [16]. A research group, Honglei Yuan et al. [17] reported the superparamagnetic Fe_3_O_4_/MWCNTs nanocomposites displayed remarkably enhanced microwave absorption characteristics in a high-frequency range (Ku-band). This research group provided an approach to improve the resonance frequency break of the Snoek limit, which considerably boosts the microwave absorption characteristics at high-frequency applications. Traditional shielding materials, such as metals and metallic composites, have drawbacks, including chemical resistance, weak flexibility, corrosion, heavy weight, and hard processibility, etc. Polymer composites as an alternative candidate for EMI shielding can provide lightweight, resistance to corrosion, flexibility, easy availability, processability, and cost-effectiveness. ZnFe_2_O_4_ is one of the most investigated spinel ferrite systems. An interesting characteristic of ZnFe_2_O_4_ is the possibility of controlling the magnetic properties with particle/crystallite size. The variation of magnetic property from paramagnetic to superparamagnetic or ferrimagnetic in ZnFe_2_O_4_ nanoparticles is attributed to cation redistribution at octahedral and tetrahedral sites [18]. The mixed cation distribution in nanosized ZnFe_2_O_4_ spinel ferrite also depends on the synthesis method [19]. Quite a lot of synthesis approaches have been utilized for the preparation of nanoparticles and their nanocomposites [20]. Sonochemistry is an innovative and potent synthesis methodology for the development of nanoparticles and nanocomposites. This technique affords noteworthy rewards, such as well dispersion, size reduction, particle de-agglomeration, homogenization, and emulsification, etc. [21]. The advantage of the sonochemical synthesis approach is cost-effectiveness, strong reaction rate, controllable synthesis, narrow particle size distribution, high purity, and nonpolluting [22,23]. Under extreme sonochemical synthesis conditions, nanomaterials with the required size can be designed at controlled chemical reactions and physical changes [24].

In the present work, structural and physical properties of superparamagnetic ZnFe_2_O_4_ spinel ferrite nanoparticles have been controlled by the sonochemical synthesis approach with increased sonochemical synthesis time. To the best of the authors’ knowledge, this is the first report on superparamagnetic ZnFe_2_O_4_ spinel ferrite nanoparticles-reduced graphene oxide (RGO)-polyurethane resin (PUR) nanocomposites as a shield against electromagnetic pollution. RGO exhibited high specific surface area and excellent electrical conductivity. However, non-magnetic RGO exhibited strong dielectric loss and also impedance mismatching issues. Therefore, the combination of magnetic spinel ferrite with the electrically conductive RGO can improve EMI-shielding performance with balanced impedance matching conditions. Additionally, the EMI-shielding performance can be improved with nanoparticles/nanostructures due to its excellent electromagnetic properties and high surface area. The structural and electromagnetic properties/parameters of developed superparamagnetic ZnFe_2_O_4_ nanoparticles and RGO embedded PUR-based nanocomposites were examined in detail. The confirmation of structural formation of prepared nanoparticles and nanocomposites was carried out by X-ray diffractometry (XRD), infrared spectrometry (FTIR), and Raman spectrometry. Microstructural features were studied by transmission electron microscopy (TEM) and field emission-scanning electron spectroscopy (FE-SEM). The sonochemical synthesis approach can also be used for the industrial-scale formation of various spinel ferrite nanoparticles. The developed lightweight and flexible EMI-shielding nanocomposite can find potential application in the field of portable electronics and wearable devices that need to be lightweight, thin, and flexible material.

## 2. Materials and Methods

### 2.1. Materials

Sodium hydroxide, zinc nitrate, and iron nitrate were acquired from Alfa Aesar GmbH & Co KG, Germany. Potassium permanganate and graphite flakes were procured from Sigma-Aldrich, Germany. Further, sodium nitrate was procured from Lach-Ner, the Czech Republic. The reducing agent, Vitamin C (Livsane), for the reduction of graphene oxide, was obtained from Dr. Kleine Pharma GmbH, Bielefeld, Germany. Polyurethane resin (PUR) was selected as a polymer elastomeric casting matrix for very low shrinkage and flexibility. PUR matrix was prepared by using Biresin^®^ U1404 elastomeric casting resin for mold-making from Sika Advanced Resins GmbH, Bad Urach, Germany, which has a description as a basis: two-component PUR system; Component A: Biresin^®^ U1404, isocyanate prepolymer, colorless-transparent, unfilled; Component B: Biresin^®^ U1404, amine, reddish-transparent, unfilled; Component B: Biresin^®^ U1434, amine, beige, filled. It provides product benefits, such as insensitive to moisture, very soft, high elongation at break, good tensile strength, and elasticity, with component B Biresin^®^ U1404 for shore hardness of A 40, with component B Biresin^®^ U1434 for shore hardness of A 55, and very low shrinkage. The PUR matrix for ZnFe_2_O_4_ and RGO as nanofillers was obtained in liquid form with isocyanate prepolymer (component A, Biresin^®^ U1404) and amine (component B, Biresin^®^ U1434) as a curing agent (hardener) with a density of 1.3 g/cm³; viscosity of ~3700 mPa-s; shore hardness of 55A; tear strength of 9 N/mm; tensile strength of 4 MPa; elongation at break >600%; linear shrinkage internal <0.02%.

### 2.2. Sonochemical Preparation of ZnFe_2_O_4_ Nanoparticles

ZnFe_2_O_4_ nanoparticles were synthesized by the sonochemical synthesis technique. First, 2.63 g Zn(NO_3_)_2_·6H_2_O and 7.56 g Fe(NO_3_)_3_·9H_2_O were mixed in 60 mL of deionized water. Additionally, aqueous 1.6 M sodium hydroxide (NaOH) was mixed in the above set solution with continuous stirring by a magnetic stirrer. Moreover, the attained mixed solution was placed to high-intensity ultrasonic waves for 25 min, 50 min, and 100 min with the use of the UZ SONOPULS HD 2070 Ultrasonic homogenizer (Berlin, Germany) at 70 W power and 20 kHz frequency. The achieved product was washed by utilizing deionized water and ethanol to eliminate unwanted chemical impurity. Finally, these washed nanoparticles were dried at 60 °C for 18 h. The prepared ZnFe_2_O_4_ nanoparticles were designated as ZS25, ZS50, and ZS100 according to their sonochemical synthesis time of 25 min, 50 min, and 100 min, respectively.

### 2.3. Preparation of Reduced Graphene Oxide (RGO)

First, graphene oxide (GO) was prepared from natural graphite powders by utilizing the Hummers’ method. Typically, the mixture comprised of graphite powder (1.5 g) and sodium nitrate (1.5 g) was added slowly to concentrated H_2_SO_4_ (75 mL). The beaker, which contains the above reaction mixture, was placed on an ice bath, and potassium permanganate (9 g) was added gradually within 20 min, and this mixed solution was stirred further for 30 min under an ice bath (temperature in the range of 0–5 °C). This solution was stirred for an additional 48 h at room temperature. Afterward, 138 mL of deionized water was gradually added into the above mixture and stirred for 10 min. In this achieved mixture, 420 mL warm deionized water was then poured and kept under a continuous strong stirring. Moreover, H_2_O_2_ (30 mL) was mixed into the above reaction mixture to eliminate the remaining KMnO_4_ and stirred until the color of the product transformed into bright yellow, which signaled the formation of graphite oxide from graphite. Finally, the obtained bright yellow product suspension was centrifuged and washed with ethanol and deionized water until the pH ~7 was reached. The obtained product was annealed at 60 °C in a vacuum oven for 24 h.

Vitamin C (10 g) was utilized as a reducing agent in the development of reduced graphene oxide (RGO) from 3 g of graphene oxide (GO). For this, the above-prepared GO was mixed in deionized water and additionally placed to high-intensity ultrasonic waves for 15 min with the use of UZ SONOPULS HD 2070 Ultrasonic homogenizer. Then, in this attained solution, vitamin C was added gradually, and then the obtained suspension was continuously stirred for the time of 3 h at temperature 90 °C. Additionally, the achieved product suspension was centrifuged and washed with ethanol and deionized water. Finally, the washed product was dried in a vacuum oven at 60 °C for 15 h.

### 2.4. Ultrasonic Preparation of Nanocomposites

Nanocomposites of polyurethane resin (PUR) (50 wt.%) with nanofillers (40 wt.% zinc ferrite nanoparticles and 10 wt.% RGO) were prepared. For the PUR matrix, component A and component B were used in the ratio of 100–50. For the preparation of nanocomposites, in a 25-mL beaker, isocyanate prepolymer (component A, Biresin U1404) were mixed with nanofillers (ZnFe_2_O_4_ (90%) + RGO (10%)) by using a EURO-ST-D mechanical stirrer for 30 min and then sonicated by using a UP 400S ultra probe (Hielscher Ultrasonics GmbH, Teltow, Germany) (frequency: 24 kHz, power: 400 W) for 30 min in an ice bath. Further, amine (component B) as a curing agent was mixed to the above mixture and then sonicated at another 10 min by using a UP 400S ultra probe (frequency: 24 kHz, power: 400 W). Finally, the prepared sample was closed and retained in a drying oven, where the composite material was cured at 25 °C for 5 days. Three PUR-based nanocomposite using zinc ferrite nanoparticles (ZS25, ZS50, or ZS100) and RGO as nanofillers, namely, (i) ZS25-RGO-PUR, (ii) ZS50-RGO-PUR, and (iii) ZS100-RGO-PUR, were prepared. Additionally, rectangle-shaped samples 22.86 × 10.16 × 1 mm^3^ were produced by cast molding.

### 2.5. Characterization Techniques

The sonochemically prepared zinc ferrite nanoparticles X-ray Diffraction (XRD) was performed using an X-ray powder diffraction from Rigaku Corporation, Tokyo, Japan. The Raman spectroscopy of PUR-based nanocomposites was performed on a Raman spectrometer of Thermo Fisher Scientific, Waltham, MA, USA. XPS study of graphene oxide and reduced graphene oxide was performed on an X-ray photoelectron spectroscope of Kratos Analytical Ltd. (Manchester, UK). The FTIR spectroscopy of ZnFe_2_O_4_ nanoparticles and PUR-based nanocomposites was performed on Nicolet 6700 (Thermo Scientific, Waltham, MA, USA). The high-resolution transmission electron microscope (JEOL JEM 2100) (JEOL, Peabody, MA, USA) was utilized to investigate the morphology and lattice fringes of ZnFe_2_O_4_ nanoparticles. The surface morphology and structure of the PUR nanocomposites were investigated with an FE-SEM of FEI NanoSEM450 (The Netherland, FEI Company). Magnetic hysteresis curves of sonochemically prepared superparamagnetic ZnFe_2_O_4_ nanoparticles were studied utilizing a VSM 7407, Lake Shore, Westerville, OH, USA. ZFC and FC temperature-dependent magnetization study of the sonochemically prepared zinc ferrite nanoparticles were investigated using a SQUID magnetometer of Quantum Design MPMS XL-7. The electromagnetic interference shielding effectiveness of the developed PUR-based nanocomposite with zinc ferrite nanoparticles and RGO as nanofillers was studied by using a vector network analyzer (Agilent N5230A, Agilent Technologies, Santa Clara, CA, USA) in 8.2–12.4 GHz (X band).

## 3. Results

### 3.1. X-ray Diffraction Study

**Figure 1:**
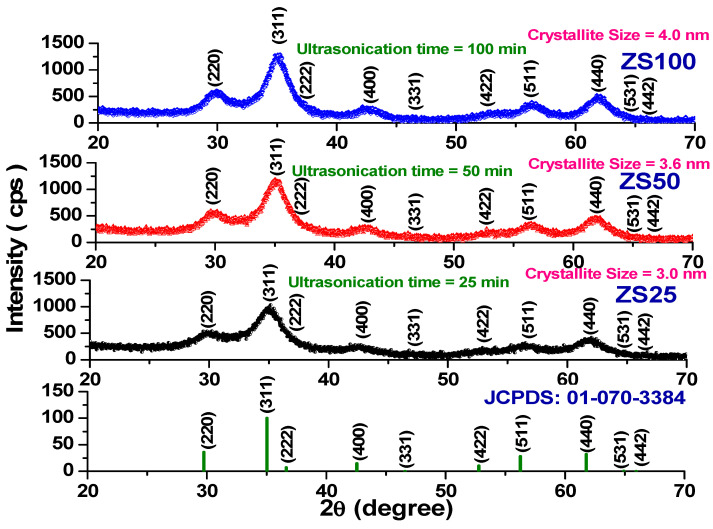
X-ray diffraction pattern of sonochemically prepared ZnFe_2_O_4_ spinel ferrite nanoparticles.

The X-ray diffraction pattern of the sonochemically synthesized ZnFe_2_O_4_ spinel ferrite nanoparticles at sonication times of 25 min, 50 min, and 100 min is displayed in Figure 1. The observed diffraction peaks correspond to the reflection of (220), (311), (222), (400), (331), (422), (511), (440), (531), and (442) planes of an Fd$\bar{3}$m spinel crystal structure. Additionally, there is no presence of an impurity peak, which designates the high purity of spinel ferrite material. It is worth noting that as the sonication synthesis time increased, the intensity of diffraction peaks increased, and the width of the diffraction peak decreased, which suggests grain growth with an increase of sonication time. The average crystallite size of synthesized ZnFe_2_O_4_ nanoparticles was studied by utilizing the Debye–Scherrer equation [25]:

(1) $$D=\frac{\left(0.9\right)\;\lambda }{\beta \cos\theta }$$

Herein, λ, β, and θ are the wavelength of X-ray, the full-width at half maximum (FWHM), and the Bragg angle, respectively. The average crystallite size increases from 3.0 nm to 4.0 nm with an increase in sonication time, as shown in Table 1. The growth of spinel ferrite nanocrystals was associated with an increase in ultrasonic time [26].

The lattice parameter was determined by utilizing the following relation [25]:

(2) $$a^{2}=\frac{\lambda^{2}{\left(h^{2}+k^{2}+l^{2}\right)}^{1/2}}{4\sin^{2}\theta }$$

Herein, θ is the Bragg angle, and (hkl) are the Miller indices of the planes. The lattice parameter increases from 7.219 Å to 7.248 Å with an increase in sonication time from 25 min to 100 min, as shown in Table 1. The observed increase in the lattice constant with sonication time follows Vegard’s law [27]. Generally, the lattice constant in the case of spinel ferrite correlates with microstructure, ordering/reordering of cations, valence states, and defects, etc. [28]. In the present work, the variation in the lattice constant can be attributed to changes in microstructure and ultrasonic-activated ordering/reordering of cations in ZnFe_2_O_4_ spinel ferrite nanoparticles.

The X-ray density (d_x_) of prepared spinel ferrite nanoparticles is evaluated by the following relation [25]:

(3) $$d_{x}=\frac{\mathrm{ZM}}{\mathrm{NV}}$$

Herein, Z, M, N, and V are the number of the nearest neighbor, the molecular weight, the Avogadro number, and the volume of the unit cell (V = a^3^), respectively. The evaluated value of the X-ray density was 8.51 g/cm^3^, 8.42 g/cm^3^, and 8.41 g/cm^3^ for ZS25, ZS50, and ZS100 samples, respectively (Table 1). Thus, an increase of sonication time to 25 min, 50 min, and 100 min decreased the density of the prepared spinel ferrite nanoparticles.

Additionally, structural parameters, such as ionic radii, hopping length for the octahedral and tetrahedral site, tetrahedral and octahedral bond length, tetrahedral edge, and the shared and unshared octahedral edge, for prepared ZnFe_2_O_4_ nanoparticles were assessed [29,30]. The variation in these parameters with sonication times of 25 min, 50 min, and 100 min was noticed, as mentioned in Table 1 and Table 2. The increase in ionic radii, hopping length for the octahedral and tetrahedral site, tetrahedral and octahedral bond length, tetrahedral edge, and the shared and unshared octahedral edge for prepared ZnFe_2_O_4_ nanoparticles with an increase of sonication time was noticed. Microstructure and ultrasonic-activated ordering/reordering of cations in ZnFe_2_O_4_ nanoparticles was associated with an increase in sonication time, which can affect the physical properties of the material [31].

### 3.2. TEM Study

**Figure 2:**
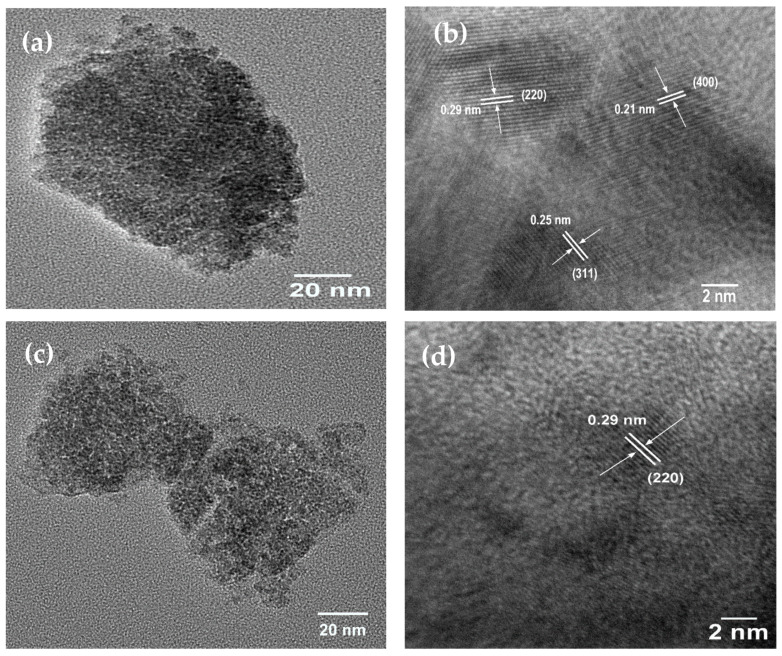

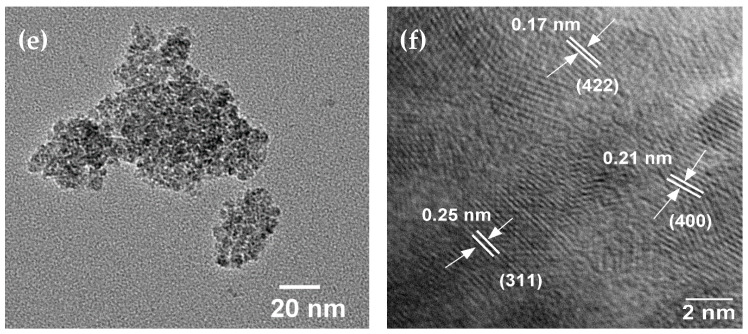
(**a**) TEM image of ZS25, (**b**) HRTEM image of ZS25, (**c**) TEM image of ZS50, (**d**) HRTEM image of ZS50, (**e**) TEM image of ZS100, and (**f**) HRTEM image of ZS100.

TEM measurements were carried out to investigate the structural features of prepared ZnFe_2_O_4_ spinel ferrite nanoparticles. Figure 2 represents TEM and HRTEM images of prepared nanoparticles, namely ZS25, ZS50, and ZS100. The TEM image of ZS25 is depicted in Figure 2a, which shows particles in the range of 2–4.5 nm (Figure S1 in supplementary material). The HRTEM image of ZS25 is shown in Figure 2b, which displays the lattice of (220) planes (d spacing 0.29 nm), (311) planes (d spacing 0.25 nm), and (400) planes (d spacing 0.21 nm) of ZnFe_2_O_4_ spinel ferrite [32]. Further, Figure 2c depicts a low-resolution TEM image of the ZS50 sample, which illustrated that the product consisted of particles with sizes of 2.5–5 nm. Figure 2d shows lattice fringes with an interplanar spacing of 0.29 nm, which is consistent with (220) planes of spinel ferrite. Additionally, the TEM image of ZS100 is depicted in Figure 2e, which demonstrated that the prepared nanoparticles exhibited size 3–12 nm. Figure 2f depicts the HRTEM image of ZS100. The investigation of the HRTEM image depicts the interplanar spacing of 0.25 nm, 0.21 nm, and 0.17 nm of lattice fringes corresponding to (311), (400), and (422) plane of ZnFe_2_O_4_ spinel ferrite.

### 3.3. FE-SEM Study

**Figure 3:**
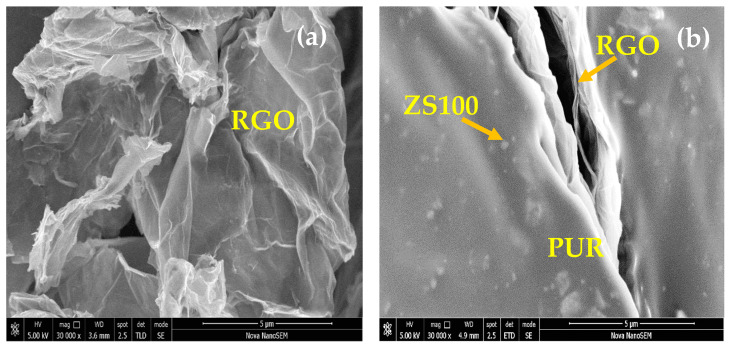

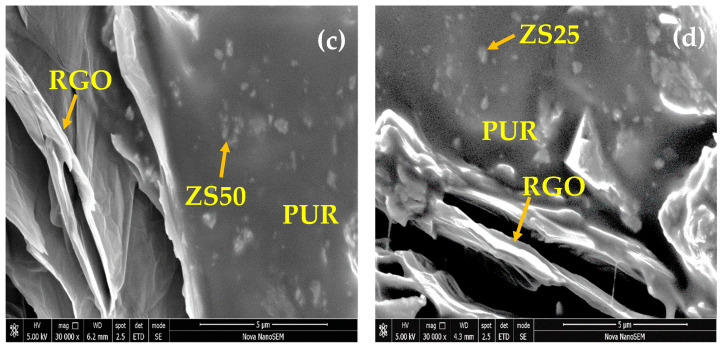
FE-SEM image of RGO (**a**), and FE-SEM image of the fracture surface of ZS100-RGO-PUR (**b**), ZS50-RGO-PUR (**c**), and ZS25-RGO-PUR (**d**).

Figure 3 depicts the typical SEM image of RGO and prepared polyurethane resin-based nanocomposites. Wrinkled and curled graphene sheets can be noticed in Figure 3a. Further, the presence of RGO and prepared ZnFe_2_O_4_ nanoparticles in polyurethane resin can be noticed in SEM images of the surfaces of the PUR-based nanocomposites, as shown in Figure 3b–d. The increase in the thickness of RGO may be due to the agglomeration of RGO during the processing and formation of polymer nanocomposite [33].

### 3.4. X-ray Photoelectron Spectroscopy

**Figure 4:**
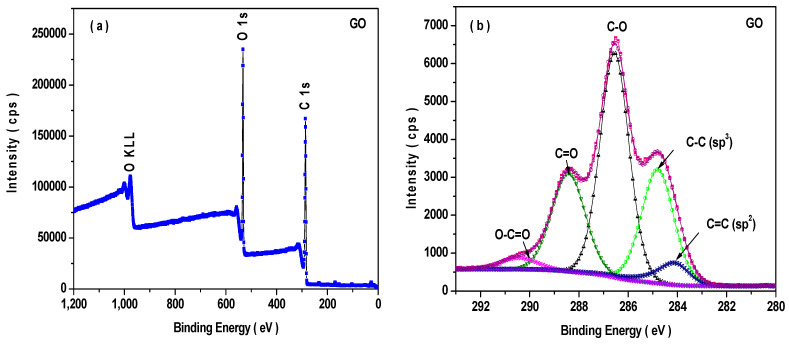

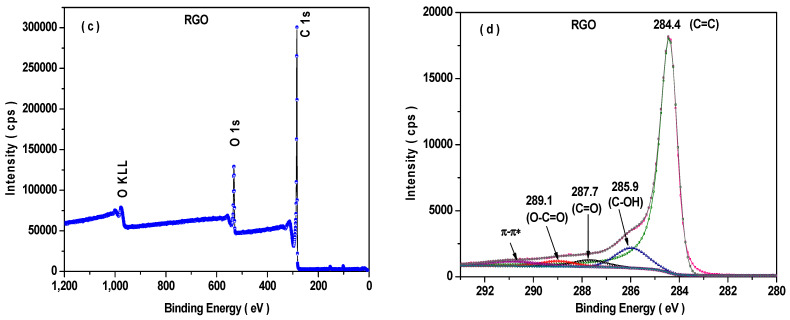
XPS spectra of GO and RGO: (**a**) survey spectrum of GO, (**b**) C1s spectrum of GO, (**c**) survey spectrum of RGO, and (**d**) C1s spectrum of RGO.

The prepared GO and RGO were examined by X-ray photoelectron spectroscopy (XPS). Figure 4 shows the XPS spectra of prepared graphene oxide (GO) and reduced graphene oxide (RGO). Figure 4a,c signifies the survey scan spectra of GO and RGO, which display the existence of carbon and oxygen. Figure 4b depicts the high-resolution XPS spectra of the C 1s region for GO. The deconvoluted C 1s peak displays the peak binding energy of 284.1 eV, 284.7 eV, 286.5 eV, 288.4 eV, and 290.0 eV, which resembles C=C (sp^2^ carbon), C-C (sp^3^ carbon), C-O, C=O, and O-C=O bonds, respectively [34]. Additionally, Figure 4d denotes the high-resolution XPS spectra of C 1s for RGO. It displays the peak binding energy of 284.4 eV, 285.9 eV, 287.7 eV, 289.1 eV, and 290.6 eV related to C=C, C-OH, C=O, O-C=O, and π-π* satellite bonds, respectively [35]. The XPS investigation demonstrated that after reduction treatment, the functional group of GO is reduced, and the sp^3^ carbon is altered to sp^2^ carbon.

### 3.5. Raman Spectroscopy

**Figure 5:**
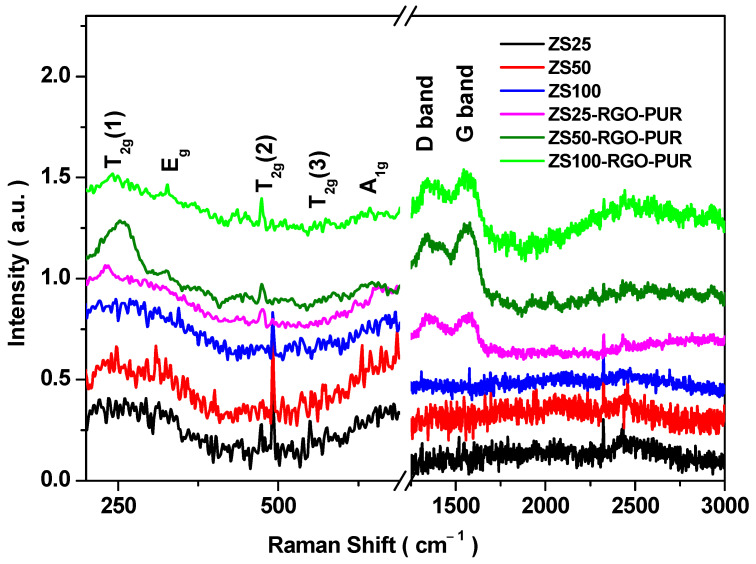
Raman spectra of ZS25, ZS50, ZS100, ZS25-RGO-PUR, ZS50-RGO-PUR, and ZS100-RGO-PUR samples.

The Raman spectroscopy examined the structural properties of synthesized nanoparticles and PUR-based nanocomposites. Figure 5 shows the Raman spectra of prepared nanoparticles, namely ZS25, ZS50, ZS100, and nanocomposites designated as ZS25-RGO-PUR, ZS50-RGO-PUR, and ZS100-RGO-PUR. Two characteristics feature Raman peaks of reduced graphene oxide, located at 1345 cm^−1^ (D band) and 1556 cm^−1^ (G band). The observed D band at 1345 cm^−1^ is ascribed to the existence of sp^3^ defects within the reduced graphene oxide sheets, whereas the G band at 1556 cm^−1^ is attributed to the E_2g_ phonon mode of in-plane sp^2^ carbon atoms [36]. Further, the observed Raman bands at ~240 cm^−1^, ~330–340 cm^−1^, ~470–490 cm^−1^, ~570 cm^−1^, and ~650 cm^−1^ were ascribed to T_2g_(1), E_g_, T_2g_(2), T_2g_(3), and A_1g_ modes for spinel ferrite ZnFe_2_O_4_ nanoparticles [37].

### 3.6. FTIR Spectroscopy

**Figure 6:**
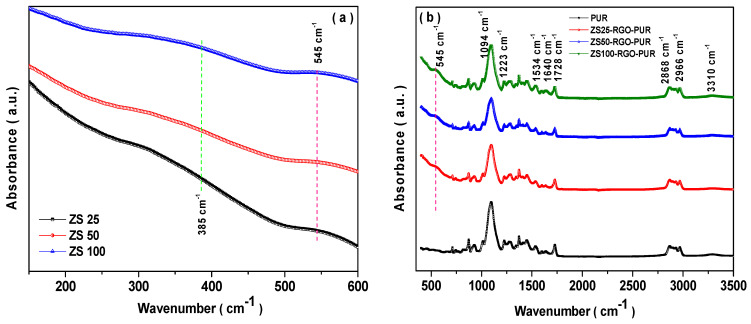
(**a**) FTIR spectra of ZS25, ZS50, and ZS100; (**b**) FTIR spectra of PUR, ZS25-RGO-PUR, ZS50-RGO-PUR, and ZS100-RGO-PUR samples.

FTIR spectroscopy is an outstanding complementary characterization tool for Raman spectroscopy characterization of nanocomposites. Figure 6a represents the FTIR spectra of prepared ZnFe_2_O_4_ nanoparticles. Two absorption bands at ~545 cm^−1^ and ~385 cm^−1^ were noted. The absorption band ~545 cm^−1^ can be ascribed to the tetrahedral Zn^2+^ (Zn-O) stretching vibration for the ZnFe_2_O_4_ spinel ferrite crystal structure. The band ~385 cm^−1^ can be assigned to the octahedral Fe^3+^ (Fe-O) stretching vibration [38]. Additionally, Figure 6b displays the FTIR spectra of pure polyurethane resin (PUR) and its nanocomposites. The presence of an absorption band ~545 cm^−1^ confirms the existence of ZnFe_2_O_4_ in the developed nanocomposite. Furthermore, Figure 6b demonstrated other absorption bands related to main characteristics peaks for polyurethane, which was observed at ~3310 cm^−1^, 2966 cm^−1^, 2868 cm^−1^, 1728 cm^−1^, 1640 cm^−1^, 1534 cm^−1^, 1223 cm^−1^, and 1094 cm^−1^. The absorption band ~3310 cm^−1^ can be ascribed to the stretching vibration of the N-H group [39]. The bands at 2966 cm^−1^ and 2868 cm^−1^ can be attributed to the non-symmetric and symmetric stretching vibration of CH_2_. Further, the absorption bands at 1728 cm^−1^, 1223 cm^−1^, and 1094 cm^−1^ can be attributed to carbonyl (C=O), aromatic C-O stretching vibration, and C-O-C non-symmetric stretching vibration. Additionally, the band ~1640 cm^−1^ can be ascribed as the abundance of amide I bands [40]. In addition, the absorption band at ~1534 cm^−1^ can be associated with the N-H bonds of the urethane group [41]. In combination with Raman and FTIR spectroscopy results, the existence of spinel ferrite ZnFe_2_O_4_ nanoparticles and reduced graphene oxide in the polyurethane matrix were verified.

### 3.7. Magnetic Properties

**Figure 7:**
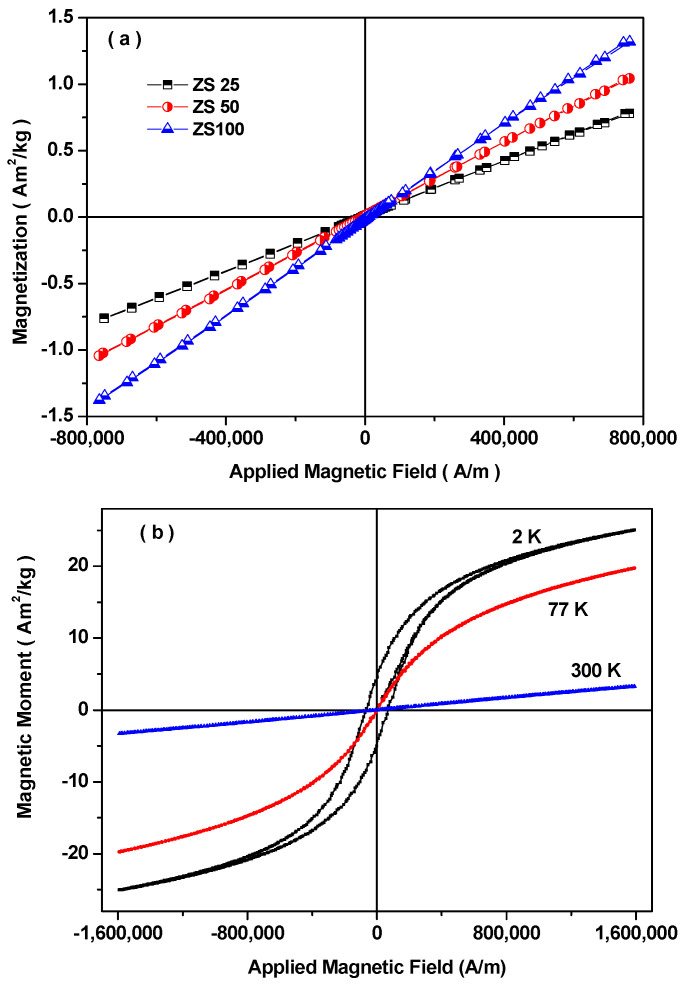

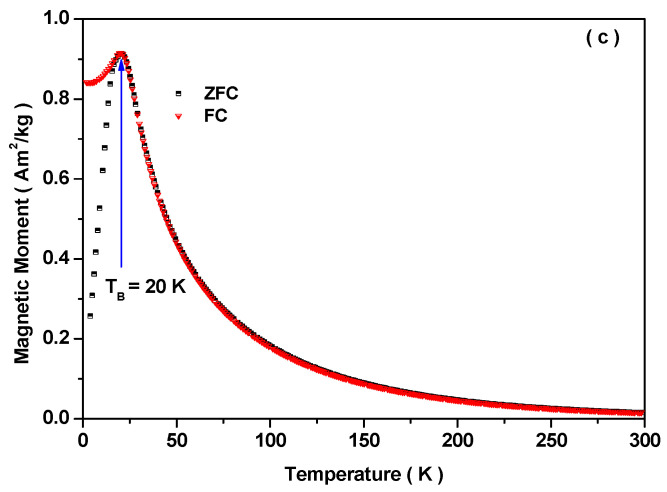
(**a**) M-H plots obtained at 300 K for ZS25, ZS50, and ZS100 samples; (**b**) M-H plots obtained at 2 K, 77 K, and 300 K for the ZS25 sample; (**c**) FC-ZFC curves for the ZS25 sample.

The magnetic characteristics of sonochemically synthesized spinel ferrite ZnFe_2_O_4_ nanoparticles were examined. Figure 7a depicts magnetic hysteresis curves of synthesized ZnFe_2_O_4_ nanoparticles at different sonication times of 25 min, 50 min, and 100 min. The prepared spinel ferrite nanoparticles exhibit zero remanent and zero coercivity, which is associated with superparamagnetic characteristics. The saturation magnetization value at the applied magnetic field of 800 kA/m was 0.78 Am^2^/kg, 1.05 Am^2^/kg, and 1.33 Am^2^/kg for ZS25, ZS50, and ZS100, respectively [42]. The reduced saturation magnetization of ZS25 is associated with the existence of a magnetically dead or anti-ferromagnetic layer on the border of the nanoparticle [43]. The observation of superparamagnetism or ferrimagnetism characteristics at room temperature in nanosized ZnFe_2_O_4_ spinel ferrite has been attributed to cation redistribution between Zn^2+^ ions at the tetrahedral sites and Fe^3+^ ions at the octahedral sites [44,45]. Further, Figure 7b depicts magnetic hysteresis curves of prepared nanoparticle sample ZS25 at various temperatures 2 K, 77 K, and 300 K. At room temperature (300 K) and 77 K, the coercivity and remanent are zero for the ZS25 sample; however, it exhibits coercivity (67 kA/m) and remanent value (5 Am^2^/kg) at 2 K, which indicates superparamagnetic behavior of prepared ZS25 sample [46,47].

The temperature dependences of zero-field cooled (ZFC) and field-cooled (FC) of prepared nanoparticle sample ZS25 were also measured in wide temperature interval 2–300 K for magnetic field 7.96 kA/m. The ZFC and FC investigation is utilized to define the blocking temperature. Figure 7c displays the irreversibility of ZFC and FC curves and the occurrence of a maximum in ZFC curves. ZFC-FC curve display irreversibility characteristic due to the blocking/freezing and unblocking mechanism of the magnetic moment of magnetic nanoparticles [48]. The rise of ZFC magnetization with temperature is associated with an unblocking progression [49]. The ZFC and FC measurements for the ZS25 sample display the blocking temperature of 20 K. The prepared ZS25 sample displays ferromagnetic behavior below 20 K and superparamagnetic above 20 K. Above the blocking temperature, the magnetic moment of nanoparticles freely fluctuates in the applied magnetic field, which leads to superparamagnetism.

Nevertheless, below the blocking temperature, the magnetic moment of each nanoparticle is blocked/freeze in the applied magnetic field direction, and the magnetic hysteresis with coercivity value 67 kA/m was noticed, as presented in Figure 7b. The blocking temperature depends on various factors such as effective anisotropy constant, magnetic coupling, particle size, applied magnetic field, etc. [50,51]. A research group, Qi Chen et al. [52], observed that the blocking temperature increases with the increase in particle size of MgFe_2_O_4_ nanoparticles. Further, Chao Liu et al. [53] reported the increase in the blocking temperature from 20 to 250 K with the increase in the size of the MnFe_2_O_4_ nanoparticles from 4.4 to 13.5 nm.

It is well-known that the saturation magnetization (M_s_) and coercivity (H_c_) of the electromagnetic wave absorber material are the most important factor to influence the magnetic loss of the electromagnetic wave shielding material [54]. In general, for the application of electromagnetic interference shielding, an initial permeability (µ_i_) signifies strong magnetic loss capacity of electromagnetic wave absorber material, which can be expressed as [55]:

(4) $${µ}_{i}=\frac{M_{S}^{2}}{{\mathrm{akH}}_{c}M_{S}+b\lambda \xi }$$

where a and b are constants, which have a dependence on the material. In the above relation, λ, ξ, and k are the magnetostriction constant, elastic strain parameter of the crystal, and proportionality coefficient, respectively [56]. The above equation signifies that the lower H_c_ and higher M_s_ are supportive of the value of µ_i_ increasing and, consequently, the performance of electromagnetic wave absorption enhancing [57]. Superparamagnetic nanoparticles that exhibited zero coercivity and higher saturation magnetization could be high-performance electromagnetic interference shielding material. In the VSM study, it is noticed the ZS100 sample exhibited a high value of magnetization as compared to ZS25 and ZS100; therefore, the permeability of nanocomposites containing ZS100 would be higher. Further, this suggests that a nanocomposite based on ZS100 could have higher electromagnetic shielding performance as compared to ZS25 and ZS50.

### 3.8. Electromagnetic Interference Shielding Effectiveness (EMI SE) Study

The total electromagnetic interference (EMI) shielding effectiveness (SE_T_) can be ascribed by the logarithmic ratio between the incoming power (P_in_) and outgoing power (P_out_) of electromagnetic radiation [58]:

(5) $${\mathrm{SE}}_{T}=10\log\left(P_{\mathrm{in}}/P_{\mathrm{out}}\right)={\mathrm{SE}}_{A}+{\mathrm{SE}}_{R}+{\mathrm{SE}}_{\mathrm{MR}}$$

Herein, SE_A_, SE_R_, SE_MR_, and SE_T_ are the absorption, reflection, multiple reflections, and total EMI shielding, respectively. In general, SE_MR_ can be ignored when SE_T_ is larger than 10 dB [58]. Hence, SE_T_ can be expressed as:

(6) $${\mathrm{SE}}_{T}={\mathrm{SE}}_{A}+{\mathrm{SE}}_{R}$$

SE_A_ in dB can be expressed as [4]:

(7) $${\mathrm{SE}}_{A}=-8.68t\frac{\sqrt{\left({fµ}_{r}\sigma_{T}\right)}}{2}$$

where f is the frequency of the electromagnetic wave; $\sigma_{T}$ is the total conductivity (S/cm) of shielding material; (${µ}_{r}$) is the complex permeability of shielding material. It can be noticed that SE_A_ is directly proportional to the conductivity ($\sigma_{T}$) and permeability (${µ}_{r}$) of the shielding material. Further, SE_R_ can be expressed as [4]:

(8) $${\mathrm{SE}}_{R}=-10\log_{10}\left(\frac{\sigma_{T}}{{µ}_{r}}\right)$$

It can be noticed from the above expression that SE_R_ is the function of the ratio of $\sigma_{T}$ and ${µ}_{r}$. Furthermore, SE_MR_ can be expressed as [4]:

(9) $${\mathrm{SE}}_{\mathrm{MR}}=20\log_{10}\left(1-10^{\frac{{\mathrm{SE}}_{A}}{10}}\right)$$

SE_MR_ can be neglected at SE_A_ ≥ 10 dB.

**Figure 8:**
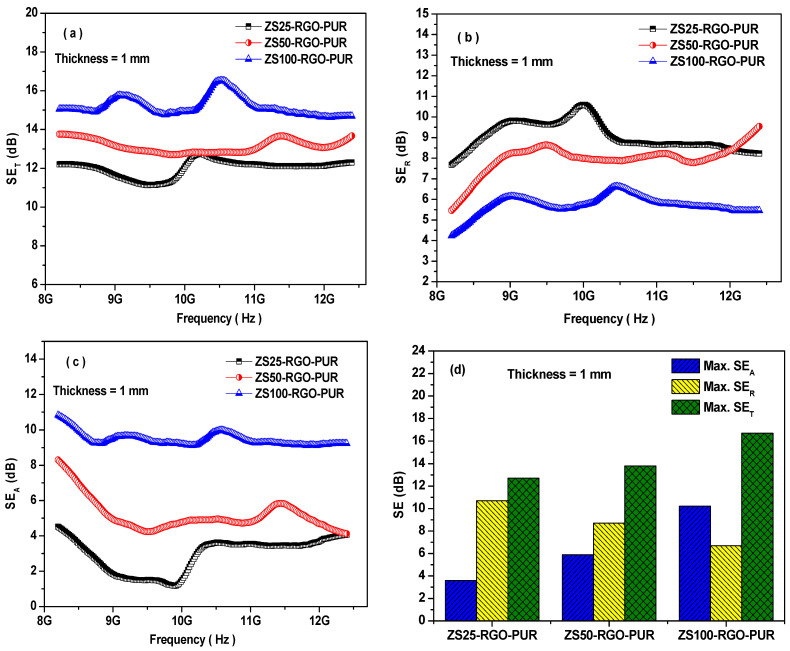
The variation of EMI-shielding effectiveness (**a**) SE_T_, (**b**) SE_R_, and (**c**) SE_A_ of superparamagnetic ZnFe_2_O_4_ and RGO-based PUR epoxy nanocomposites at the thickness of 1 mm; (**d**) a comparison of the maximum values of SE_T_, SE_R_, and SE_A_.

Figure 8 depicts the EMI-shielding performance of prepared nanocomposites based on the polyurethane matrix with sonochemically prepared superparamagnetic ZnFe_2_O_4_ nanoparticles and RGO as nanofillers. The maximum total shielding effectiveness (SE_T_) value for prepared nanocomposites of thickness 1 mm in the frequency range of 8.2–12.4 GHz was 12.7 dB, 13.8 dB, and 16.7 dB, for ZS25-RGO-PUR, ZS50-RGO-PUR, and ZS100-RGO-PUR, respectively. The EMI SE_T_ value increases with an increase in the size of utilized superparamagnetic ZnFe_2_O_4_ spinel ferrite nanoparticles in developed nanocomposites. The maximum SE_A_ value was 3.6 dB, 5.9 dB, and 10.2 dB for prepared nanocomposites ZS25-RGO-PUR, ZS50-RGO-PUR, and ZS100-RGO-PUR, respectively. The higher SE_A_ value of the ZS100-RGO-PUR sample indicates that this nanocomposite exhibits higher electrical conductivity and higher magnetic permeability. Additionally, the maximum SE_R_ value was noticed to be 10.7 dB, 8.7 dB, and 6.7 dB for developed composite material ZS25-RGO-PUR, ZS50-RGO-PUR, and ZS100-RGO-PUR, respectively.

In recent years, a research group, Xiaogu Huang et al. [59], reported the minimum reflection loss of −3.8 dB, −5 dB, and −8.1 dB for ZnFe_2_O_4_ nanoparticles, nanorods, nanofibers, respectively. Another research group, Chang Sun et al. [60], noticed a minimum reflection loss value of −10.4 dB at 17.7 GHz with a matching thickness of 16.7 mm for LiFeO_2_/ZnFe_2_O_4_ composite. Further, Ruiwen Shu et al. [61] noticed the value of the minimum reflection loss of −12.0 dB with a thickness of 4.5 mm for hybrid nanocomposites of reduced graphene oxide/zinc ferrite fabricated by a facile one-pot hydrothermal strategy. Furthermore, Junsheng Xue et al. [62] reported microwave absorption characteristics of ZnFe_2_O_4_ nanoparticles of 130 nm and noticed minimum reflection loss of ZnFe_2_O_4_/paraffin of −23.4 dB at 17.9 GHz for higher thickness around 9 mm. Moreover, Wei Ma et al. [63] reported that the reduced graphene oxide/zinc ferrite/nickel nanohybrids exhibited the reflection loss (RL) of −22.57 dB at 4.21 GHz with a thickness of 2.5 mm. The electromagnetic interference shielding or microwave absorption characteristics of ZnFe_2_O_4_ nanoparticles depend on various factors, including size, morphology, compositions, the thickness of the sample, and composite matrix, etc. [64,65,66].

### 3.9. Electromagnetic Properties and Parameters

**Figure 9:**
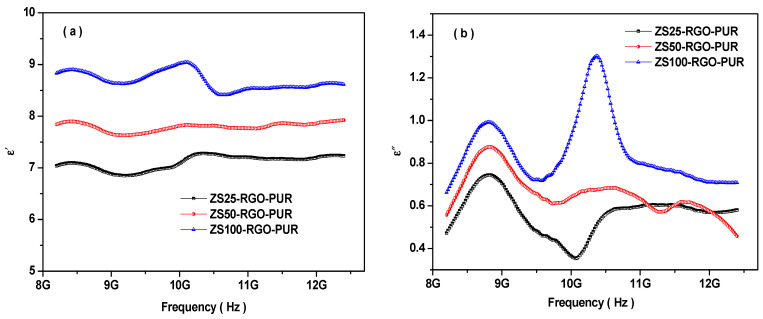

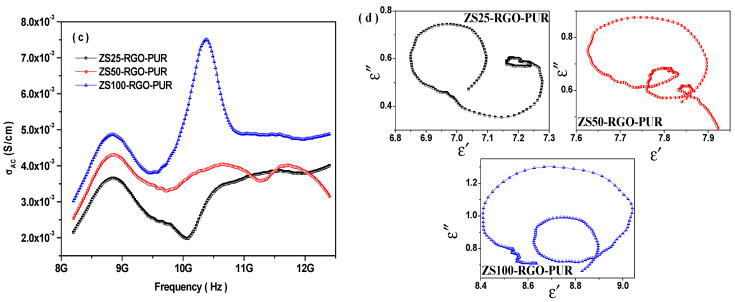
(**a**) Frequency dependence of the real permittivity (ε′), (**b**) frequency dependence of the imaginary permittivity (ε”), (**c**) frequency dependence of the ac conductivity, and (**d**) Cole–Cole plots of nanocomposites.

To elaborate more on the shielding characteristics of prepared superparamagnetic ZnFe_2_O_4_ and RGO-based PUR nanocomposites, complex permittivity and permeability of the nanocomposites were investigated. The real permittivity (ε′) implies the storage ability of electrical energy, while the imaginary permittivity (ε″) signifies energy dissipation. Figure 9a represents the frequency dependence real permittivity for developed nanocomposites in the 8.2–12.4 GHz frequency range. The value of ε′ is in the ranges of 6.8 to 7.3, 7.5 to 7.9, 8.4 to 9.1 for developed nanocomposites ZS25-RGO-PUR, ZS50-RGO-PUR, and ZS100-RGO-PUR, respectively. Figure 9b represents the frequency dependence imaginary permittivity for nanocomposites in the 8.2–12.4 GHz frequency range. The value of ε″ is in the range of 0.35 to 0.74, 0.55 to 0.88, 0.65 to 1.31 for ZS25-RGO-PUR, ZS50-RGO-PUR, and ZS100-RGO-PUR, respectively. The complex permittivity is the result of the polarizability of the nanocomposite material associated with the dipolar and electric polarization, initiated by an EM wave [5,67]. The input to the space charge polarization acts because of the heterogeneity of the nanocomposite material. In heterogeneous dielectric materials, there is an accumulation of virtual charges on the interfaces of two mediums with different dielectric constants and conductivities, which lead to interfacial polarization and is called Maxwell–Wagner polarization [68,69]. It can be observed that the values of ε′ and ε″ are increased with the increase of the size of superparamagnetic ZnFe_2_O_4_ nanoparticles in prepared nanocomposites.

The relation between electrical conductivity (σ_AC_) and imaginary permittivity (ε″) can be stated as [70]:

(10) $$\sigma_{\mathrm{AC}}=\varepsilon_{o}\varepsilon^{\prime \prime }2\pi f$$

Herein, ε_o_ is the dielectric constant of free space; f is the frequency of the electromagnetic wave. The above relation signifies that the electrical conductivity will increase with an increase in the value of imaginary permittivity. Therefore, the enhanced value of the complex permittivity can be associated with the increase in the electrical conductivity of the prepared nanocomposites with an increase in the size of embedded superparamagnetic ferrite nanoparticles. Figure 9c represents the change in electrical conductivity with the frequency of prepared nanocomposites. The electrical conductivity is in the range of 1.9 × 10^−3^ to 3.9 × 10^−3^ S/cm, 2.5 × 10^−3^ to 4.3 × 10^−3^ S/cm, 2.9 × 10^−3^ to 7.5 × 10^−3^ S/cm for ZS25-RGO-PUR, ZS50-RGO-PUR, and ZS100-RGO-PUR, respectively.

Further, in reported literature by other researchers, the Debye theory is generally utilized to clarify the relaxation process of dipoles [71,72]. According to the Debye theory for dielectric loss characteristics, the real permittivity (ε′) and imaginary permittivity (ε″) can be written as [73]:

(11) $$\begin{array}{c} \varepsilon^{\prime }=\varepsilon_{\infty }+\frac{\varepsilon_{s}-\varepsilon_{\infty }}{1+{\left(\omega \tau \right)}^{2}}\\ \varepsilon^{\prime \prime }=\varepsilon_{\mathrm{relax}}^{\prime \prime }+\varepsilon_{\sigma }^{\prime \prime }=\frac{\varepsilon_{s}-\varepsilon_{\infty }}{1+{\left(\omega \tau \right)}^{2}}\omega \tau +\frac{\sigma }{{\omega \varepsilon }_{o}} \end{array}$$

Herein, ε_s_ and ε_∞_ are the static and infinite permittivity; ω = 2πf is the angular frequency; τ is the relaxation time; σ is the conductivity. It can be seen from the above relation that the ε′ and ε″ are the functions of ωτ. Hence, both the ε′ and ε″ are mutually dependent on one another. A relationship between ε′ and ε″ can be inferred after ignoring the contribution of σ and by eliminating ωτ [74]:

(12) $${\left(\varepsilon^{\prime }-\frac{\varepsilon_{s}+\varepsilon_{\infty }}{2}\right)}^{2}+{\left(\varepsilon^{\prime \prime }\right)}^{2}={\left(\frac{\varepsilon_{s}-\varepsilon_{\infty }}{2}\right)}^{2}$$

From the above relation, it is easy to recognize that the curves of ε′ and ε″ would be a semi-circle, which is known as the Cole–Cole semicircle [75].

Figure 9d depicts the Cole–Cole plots for the developed PUR-based nanocomposites. In general, the relaxation is associated with a delay in polarization concerning the change in the electrical field. Some obvious Cole–Cole semicircles can be noticed in Figure 9d, which signifies that the relaxation contributed to the dielectric loss. Additionally, one Cole–Cole semicircle represents a Debye dipolar relaxation, and the existence of more semicircles is attributed to multiple relaxation processes [76]. These other semicircles are associated with Maxwell–Wagner relaxation, electron/ion polarization, and interfacial polarization [77]. The multiple dielectric losses were responsible for the improvement of the absorption characteristics of PUR-based nanocomposites.

**Figure 10:**
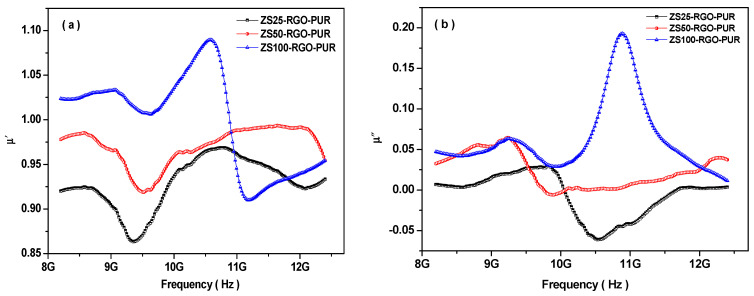

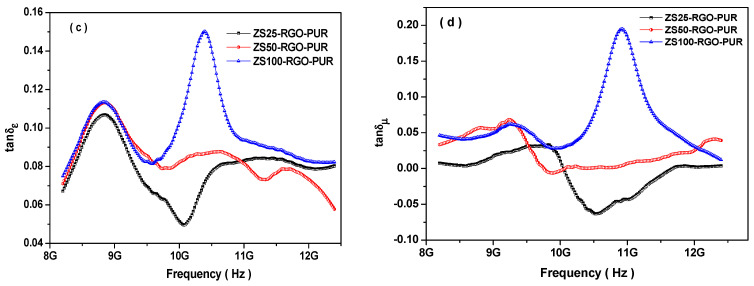
(**a**) Frequency dependence of the real permeability (µ′), (**b**) frequency dependence of the imaginary permeability (µ″), (**c**) dielectric loss tangent, and (**d**) magnetic loss tangent of nanocomposites.

It is well-known that the real permeability (µ′) represents the storage ability of magnetic energy, and the imaginary permeability (µ″) signifies the magnetic loss. Figure 10a represents the frequency dependence of the real permeability (µ′) of PUR-based nanocomposites. The µ′ is in the range of 0.86 to 0.96, 0.91 to 0.99, and 0.90 to 1.09 for nanocomposites ZS25-RGO-PUR, ZS50-RGO-PUR, and ZS100-RGO-PUR, respectively. The value of real permeability (µ′) was increased with an increase of grain size of utilized superparamagnetic ZnFe_2_O_4_ spinel ferrite nanoparticles. Further, the value of µ″ is in the range of −0.06 to 0.03, −0.01 to 0.07, and 0.03 to 0.19 for the prepared composites ZS25-RGO-PUR, ZS50-RGO-PUR, and ZS100-RGO-PUR, respectively, as shown in Figure 10b. Remarkably, it is noticed that the µ″ exhibited negative value also for some PUR-based nanocomposites, which is associated with the motion of charges [78].

Additionally, the Globus equation is expressed as [79]:

(13) $$µ\propto {\left(M_{s}^{2}D/K_{1}\right)}^{1/2}$$

This equation signifies that to get a higher complex permeability, a higher saturation magnetization (M_S_), larger grain size (D), and smaller magnetocrystalline anisotropy constant (K_1_) are needed. The increased magnetization and larger grain size of the ZS100 sample may add to the larger permeability for prepared ZS100-RGO-PUR nanocomposites, as compared with ZS25-RGO-PUR and ZS50-RGO-PUR nanocomposites.

Further, based on the following relations [80]:

(14) $$\begin{array}{c} \tan\delta_{\varepsilon }=\frac{\varepsilon^{\prime \prime }}{\varepsilon^{\prime }}\\ \tan\delta_{µ}=\frac{{µ}^{\prime \prime }}{{µ}^{\prime }} \end{array}$$

and utilizing electromagnetic parameters for ZS25-RGO-PUR, ZS50-RGO-PUR, and ZS100-RGO-PUR nanocomposites, the dielectric loss tangent (tanδ_ε_) and magnetic loss tangent (tanδ_µ_) were evaluated. Figure 10c represents dielectric loss tangent vs. frequency curves for prepared PUR-based nanocomposites. The dielectric loss tangent (tanδ_ε_) of samples ZS25-RGO-PUR, ZS50-RGO-PUR, and ZS100-RGO-PUR, fluctuated with an increase of frequency of electromagnetic wave between 0.05 to 0.10, 0.06 to 0.11, and 0.07 to 0.15, respectively. Additionally, the dielectric loss is related to dipole polarization and interfacial polarization at higher frequencies [81]. It can be also noticed that the dielectric loss (ε″) value of the ZS100-RGO-PUR sample is much higher than the other two samples (i.e., ZS25-RGO-PUR, and ZS50-RGO-PUR). The higher dielectric loss in the ZS100-RGO-PUR sample is associated with enhanced electrical conductivity and dielectric constant induced by micro-currents and polarization in nanocomposites [82].

The magnetic loss tangent variation with the frequency of an electromagnetic wave of prepared PUR-based nanocomposites is presented in Figure 10d. It can be perceived that the magnetic loss tangent fluctuated between −0.06 to 0.03, −0.01 to 0.07, and 0.03 to 0.19 for samples ZS25-RGO-PUR, ZS50-RGO-PUR, and ZS100-RGO-PUR, respectively.

**Figure 11:**
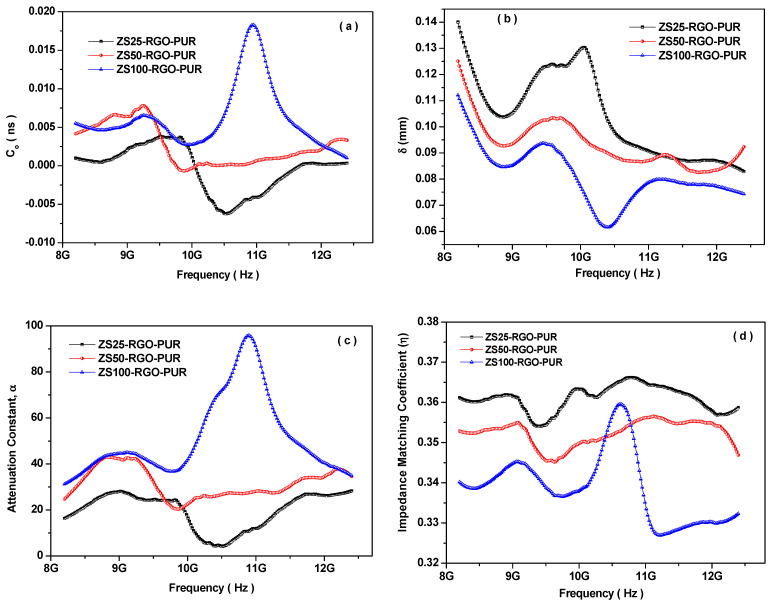
Frequency dependence of (**a**) the eddy current loss, (**b**) skin depth, (**c**) attenuation constant, and (**d**) impedance matching coefficient for prepared PUR-based nanocomposites.

It is well-known that natural resonance, exchange resonance, and eddy current are the main contributors to the magnetic loss of nanoparticles [83]. The eddy current loss can be stated by the following relation when the size of magnetic nanoparticle (D) is smaller than the skin depth (δ) [84]:

(15) $$\frac{{µ}^{\prime \prime }}{{µ}^{\prime }}\alpha \frac{{µ}^{\prime }\mathrm{fD}}{\rho }$$

where f is the electromagnetic wave frequency; ρ is the electric resistivity of the nanoparticles. Based on this above relation, C_o_ = f^−1^(µ′)^−2^µ″ should be constant, if the magnetic loss is mainly contributed from the eddy current loss. It can be seen in Figure 11a that the value C_o_ is not constant for all the prepared PUR-based nanocomposites. It signifies that the eddy current loss would not be a dominant contributor to magnetic loss.

Besides dielectric and magnetic losses, skin depth (δ) is another important factor that stimulates the absorption of electromagnetic waves. Skin depth states the distance at which the field drops to 1/e of the incident value and stated as [85]:

(16) $$\delta =1/\sqrt{\pi fµ\sigma }$$

Herein, f is the frequency; σ is the electrical conductivity; µ is the permeability. This relation signifies that skin depth reduces with an increase in frequency, permeability, and conductivity. Figure 11b depicts the frequency dependence variation of skin depth of the prepared PUR-based nanocomposites. A smaller skin depth states a stronger absorption capacity [86]. The skin depth of samples ZS25-RGO-PUR, ZS50-RGO-PUR, and ZS100-RGO-PUR fluctuated with an increase in the frequency of electromagnetic waves between 0.08 to 0.14 mm, 0.08 to 0.12 mm, and 0.06 to 0.11 mm, respectively. The prepared ZS100-RGO-PUR nanocomposite exhibits smaller skin depth and, therefore, stronger absorption.

The attenuation constant (α) is an important factor that governs the electromagnetic wave absorption capabilities of shielding nanocomposites. It can be assessed by the following relation [87]:

(17) $$\alpha =\frac{\sqrt{2}\pi f}{c}\sqrt{\left({µ}^{\prime \prime }\varepsilon^{\prime \prime }-{µ}^{\prime }\varepsilon^{\prime }\right)+\sqrt{{\left({µ}^{\prime }\varepsilon^{\prime \prime }+{µ}^{\prime \prime }\varepsilon^{\prime }\right)}^{2}+{\left({µ}^{\prime \prime }\varepsilon^{\prime \prime }-{µ}^{\prime }\varepsilon^{\prime }\right)}^{2}}}$$

**Figure 12:**
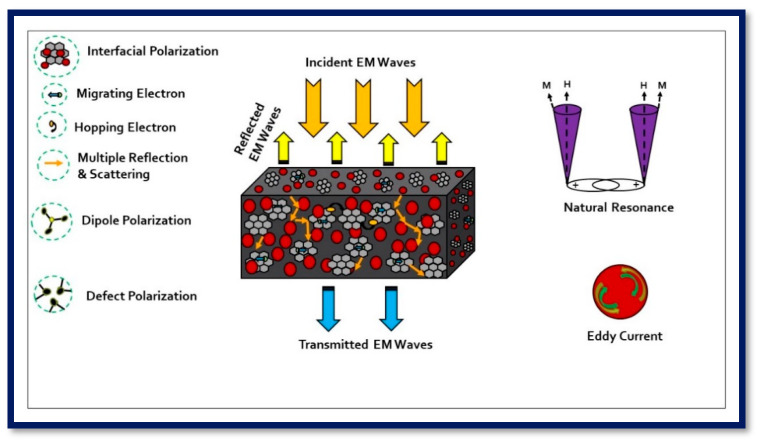
Schematic illustration of the electromagnetic interference shielding mechanism in prepared nanocomposites.

Figure 11c displays the frequency dependence variation of the attenuation constant (α) of prepared PUR-based nanocomposites. The high attenuation constant (α) value of the prepared ZS100-RGO-PUR nanocomposite compared with other samples demonstrated high absorption of electromagnetic waves. Another key factor that governs electromagnetic wave absorption is impedance matching. It is stated by the modulus of the normalized characteristic impedance (Z), which can be calculated by utilizing the following relation [88]:

(18) $$Z=\left|Z_{1}/Z_{o}\right|$$

where $Z_{1}=Z_{o}\sqrt{{µ}_{r}/\varepsilon_{r}}$; Zo is the impedance in free space; ε_r_ is the value of complex permittivity; µ_r_ is the value of complex permeability. Figure 11d shows the variation of the impedance matching coefficient for prepared PUR-based nanocomposites with frequency. The values of Z are below one, and the ZS100-RGO-PUR nanocomposite has a lower Z value compared to other samples. A high attenuation constant and moderate Z value of ZS100-RGO-PUR nanocomposite provided the high value of EMI-shielding effectiveness [89]. The schematic illustration of the electromagnetic interference shielding mechanism in the prepared nanocomposite is shown in Figure 12. When electromagnetic waves interact at the surface of the prepared nanocomposite, a part of it is reflected, another part is absorbed, and the remaining part has multiple reflections and scattering [90]. The reflection is associated with moving charge carriers interacted with electromagnetic waves [91]. The absorption signifies the dissipation of energy of the electromagnetic waves due to the interaction of electromagnetic waves with the electric and magnetic dipoles [92]. The multiple reflections are reflections at different surfaces or interfaces present due to inhomogeneity within the prepared nanocomposite. The prepared nanocomposites consisted of conductive RGO sheets and ZnFe_2_O_4_ magnetic nanoparticles not only improve impedance matching but also creates a micro-current network and attains interfacial polarization [93]. The appropriate conductivity of RGO sheets gifted the prepared nanocomposites exhibited a moderate conductivity loss. Residual functional groups and defects in RGO sheets and ZnFe_2_O_4_ originated dipole polarization and defect polarization [94]. The interaction of electromagnetic waves also creates hopping and migrating electrons across the defects of RGO sheets.

The magnetic characteristics of the superparamagnetic ZnFe_2_O_4_ nanoparticles component in the developed nanocomposites provided a degree of magnetic loss such as natural resonance and eddy current loss. It improves the impedance matching between complex permittivity and permeability, which provides well absorption condition for electromagnetic waves [95]. The improved electromagnetic wave shielding characteristics of the ZS100-RGO-PUR nanocomposite can be primarily attributed to the inclusive acts of magnetic loss, dielectric loss, and appropriate attenuation constant derived from various nanofillers in the matrix.

## 4. Conclusions

In summary, superparamagnetic ZnFe_2_O_4_ spinel ferrite nanoparticles were prepared successfully by the sonochemical synthesis approach at various ultra-sonication times of 25 min (ZS25), 50 min (ZS50), and 100 min (ZS100). The average crystallite size increased from 3.0 nm to 4.0 nm with an increase in sonication time. The lattice parameter increased from 7.219 Å to 7.248 Å with an increase in sonication time from 25 min to 100 min. The increase in ionic radii, hopping length for the octahedral and tetrahedral site, tetrahedral and octahedral bond length, tetrahedral edge, and shared and unshared octahedral edge for prepared ZnFe_2_O_4_ nanoparticles with an increase in sonication time is associated with cation redistribution in ZnFe_2_O_4_ nanoparticles with an increase in sonication time. The prepared spinel ferrite nanoparticles exhibited zero remanent and zero coercivity, which is associated with superparamagnetic characteristics. The prepared magnetic ZnFe_2_O_4_ nanoparticles (ZS25, ZS50, and ZS100) and electrically conductive reduced graphene oxide (RGO) were embedded in a polyurethane resin (PUR) matrix to develop lightweight and flexible nanocomposites for electromagnetic interference shielding application. The maximum total shielding effectiveness (SE_T_) value for developed nanocomposites of thickness 1 mm in the range of 8.2–12.4 GHz frequency was 12.7 dB, 13.8 dB, and 16.7 dB, for ZS25-RGO-PUR, ZS50-RGO-PUR, and ZS100-RGO-PUR, respectively. The higher attenuation constant (α) value of in prepared ZS100-RGO-PUR nanocomposite as compared with other samples demonstrated high absorption of electromagnetic waves. This work demonstrated an ingenious and effective strategy to develop polyurethane resin-based nanocomposites consisting of superparamagnetic ZnFe_2_O_4_ spinel ferrite nanoparticles with RGO for shielding electromagnetic pollution.

## Figures and Tables

**Table 1:** Crystallite size, Lattice Parameter, X-ray Density, and Ionic Radii (r_A_, r_B_) for the prepared ZnFe_2_O_4_ spinel ferrite nanoparticles by the sonochemical synthesis approach.

Sample	Crystallite Size (nm)	Lattice Parameter, a (Å)	X-ray Density d_x_ (g/cm^3^)	Ionic Radii r_A_ (Å)	Ionic Radii r_B_ (Å)
**ZS 25**	3.0	7.219	8.51	0.2757	1.4026
**ZS 50**	3.6	7.245	8.42	0.2816	1.4087
**ZS 100**	4.0	7.248	8.41	0.2822	1.4093

**Table 2:** Structural parameters for prepared ZnFe_2_O_4_ nanoparticles synthesized by sonochemical approach: hopping length for the octahedral and tetrahedral site, tetrahedral and octahedral bond length, tetrahedral edge, and the shared and unshared octahedral edge.

Sample	Hopping Length for Tetrahedral Site d_A_ (Å)	Hopping Length for Octahedral Site d_B_ (Å)	Tetrahedral Bond Length, d_Ax_ (Å)	Octahedral Bond Length, d_Bx_ (Å)	Tetrahedral Edge, d_AxE_ (Å)	Shared Octahedral Edge, d_BxE_ (Å)	Unshared Octahedral Edge, d_BxEU_ (Å)
**ZS 25**	3.1263	2.5526	1.6757	1.7424	2.7364	2.3688	2.5559
**ZS 50**	3.1374	2.5617	1.6816	1.7486	2.7461	2.3772	2.5650
**ZS 100**	3.1384	2.5625	1.6822	1.7491	2.7470	2.3780	2.5658

## Data Availability

Data can be available upon request from the corresponding author.

## Supplementary Materials

The following are available online at https://www.mdpi.com/article/10.3390/nano11051112/s1, Figure S1: (a) TEM image of ZS25, (b) size distribution of ZS25, (c) TEM image of ZS50, (d) size distribution of ZS50, (e) TEM image of ZS100, (f) size distribution of ZS100.
