# Supplementary material for: Superparamagnetic ZnFe2O4 Nanoparticles-Reduced Graphene Oxide-Polyurethane Resin Based Nanocomposites for Electromagnetic Interference Shielding Application
Source: Nanomaterials (Basel). 2021 Apr 25;11(5):1112. doi: 10.3390/nano11051112 (PMC8145072; doi:10.3390/nano11051112)
Supplement: Supplementary file 1 [file nanomaterials-11-01112-s001.zip › nanomaterials-1183956-supplementary.pdf]

nanomaterials

MDPI

Supplementary Material

Superparamagnetic $ZnFe_2O_4$ Nanoparticles-Reduced Graphene Oxide-Polyurethane Resin Based Nanocomposites for Electromagnetic Interference Shielding Application

Raghvendra Singh Yadav [1,*], Anju [1], Thaiskang Jamatia [1], Ivo Kuřitka [1], Jarmila Vilčáková [1], David Škoda [1], Pavel Urbánek [1], Michal Machovský [1], Milan Masař [1], Michal Urbánek [1], Lukas Kalina [2] and Jaromir Havlica [2]

[1] Centre of Polymer Systems, University Institute, Tomas Bata University in Zlín, Trida Tomase Bati 5678, 760 01 Zlín, Czech Republic; deswal@utb.cz (A.); jamatia@utb.cz (T.J.); kuritka@utb.cz (I.K.); vilcakova@utb.cz (J.V.); dskoda@utb.cz (D.Š.); urbanek@utb.cz (P.U.); machovsky@utb.cz (M.M.); masar@utb.cz (M.M.); murbanek@utb.cz (M.U.)

[2] Materials Research Centre, Brno University of Technology, Purkyňova 464/118, 61200 Brno, Czech Republic; kalina@fch.vut.cz (L.K.); havlica@fch.vut.cz (J.H.)

* Correspondence: yadav@utb.cz; Tel.: +420-576031725

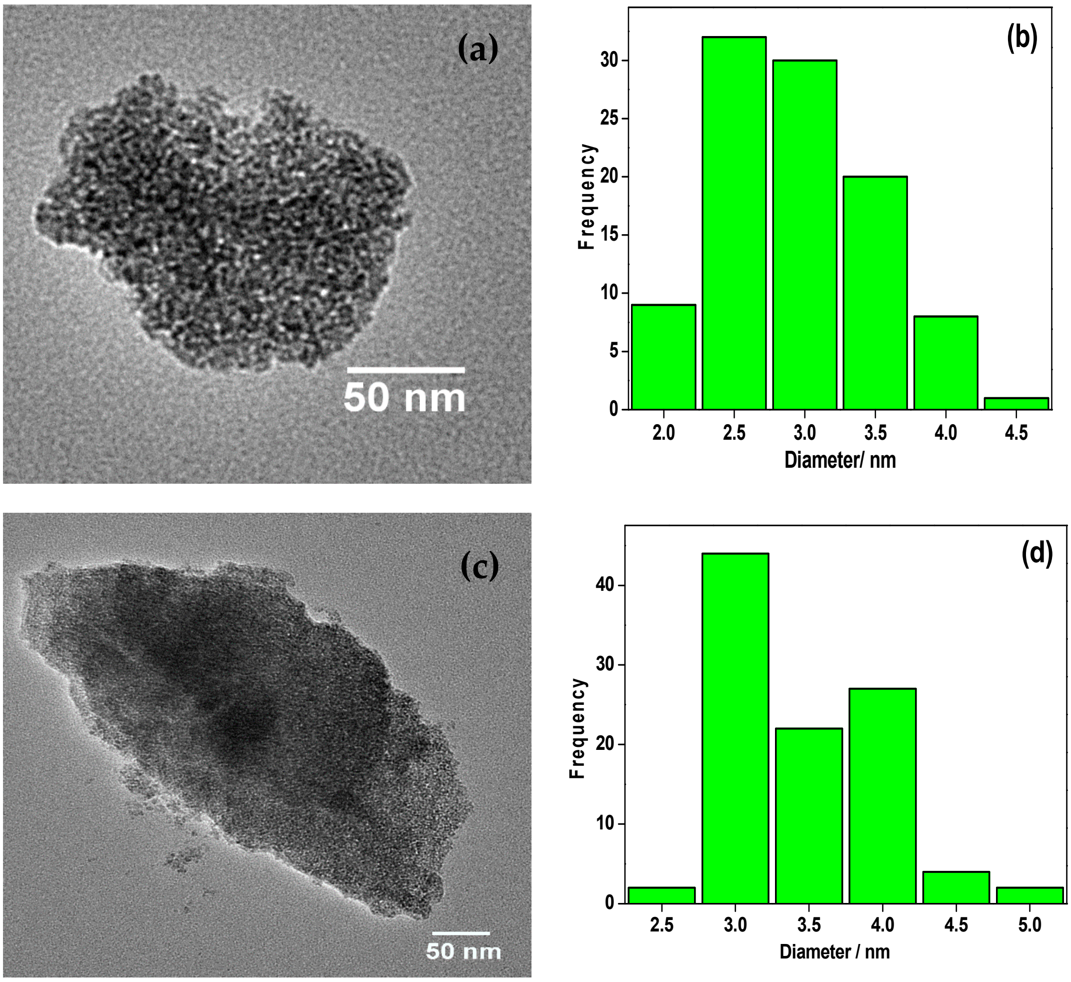

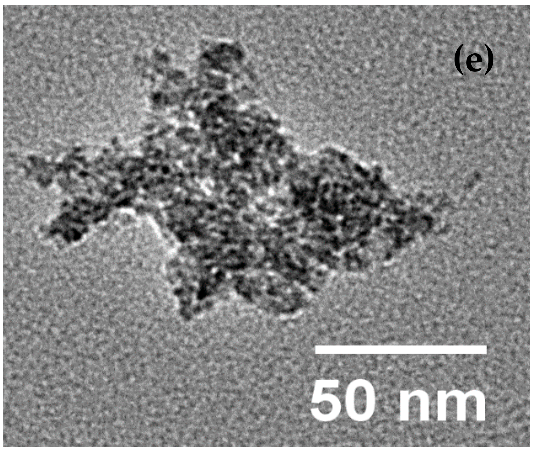

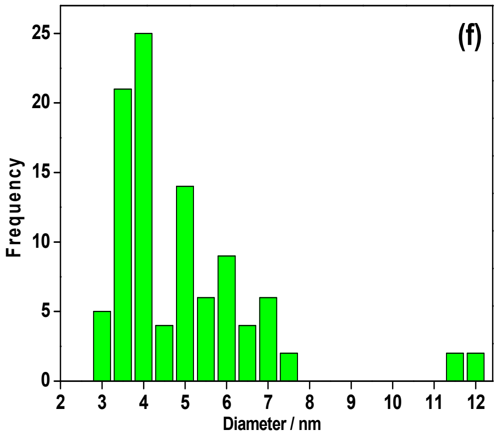

Figure S1: (**a**) TEM image of ZS25, (**b**) the size distribution of ZS25, (**c**) TEM image of ZS50, (**d**) the size distribution of ZS50, (**e**) TEM image of ZS100, and (**f**) the size distribution of ZS100.
